# Acute effects of singing on cardiovascular biomarkers

**DOI:** 10.3389/fcvm.2022.869104

**Published:** 2022-07-18

**Authors:** Kamila Somayaji, Mogen Frenkel, Luai Tabaza, Alexis Visotcky, Tanya Kruse Ruck, Ernest Kwesi Ofori, Michael E. Widlansky, Jacquelyn Kulinski

**Affiliations:** ^1^Division of Cardiology, Department of Medicine, Medical College of Wisconsin, Milwaukee, WI, United States; ^2^Department of Anesthesiology, Medical College of Wisconsin, Milwaukee, WI, United States; ^3^Division of Cardiovascular Diseases, Einstein Medical Center, Philadelphia, PA, United States; ^4^Division of Biostatistics, Medical College of Wisconsin, Institute for Health and Equity, Milwaukee, WI, United States; ^5^Department of Music, Peck School of the Arts, University of Wisconsin-Milwaukee, Milwaukee, WI, United States; ^6^Department of Physical Therapy, Whitworth University, Spokane, WA, United States

**Keywords:** singing, vascular function, endothelial function, heart rate variability (HRV), cardiac rehabilitation

## Abstract

**Background:**

Singing is a physical activity involving components of the vagal nerves manifested as changes in cardiac autonomic regulation.

**Aims:**

The aim of this pilot study is to investigate the acute effects of singing on biomarkers of cardiovascular health.

**Methods:**

Adult subjects were recruited from cardiology clinics to participate in a single 90-min study visit. Vascular function was measured at the fingertips with peripheral arterial tonometry (PAT) before and after singing to a 14-min video led by a voice expert. Heart rate variability (HRV) was measured with a chest strap sensor at baseline, during, and after singing. PAT measurements were expressed as reactive hyperemia index (RHI) and Framingham reactive hyperemia index (fRHI). Measures of HRV included root mean square of successive RR interval differences (RMSSD) and standard deviation of NN (or RR) intervals (SDNN).

**Results:**

Sixty subjects completed the study (68% female, mean age 61 ±13 years, mean BMI 32 ± 8). There was a significant increase in fRHI (1.88 ± 0.14 to 2.10 ± 0.14, *p* = 0.02) after singing with no significant change in the RHI (1.99 ± 0.10 to 2.12 ± 0.09, *p* = 0.22). There was a reduction in HRV during singing (compared to baseline) (RMSSD: 42.0 ± 5 to 32.6 ± 4, *p* = 0.004 and SDNN: 54 ± 4 to 33.5 ± 3, *p* = 0.009). HRV measures trended back toward baseline after singing.

**Conclusions:**

A short duration of singing improved vascular function acutely. Improvements were more substantial in subjects with abnormal baseline endothelial function. HRV patterns were similar to that of light-intensity exercise. Future studies should confirm favorable vascular adaptation to more sustained singing interventions.

**Clinical trial registration:**

ClinicalTrials.gov, identifer: NCT03805529.

## Introduction

Cardiovascular disease (CVD) is the leading cause of death in most developed countries (in men and women), claiming more lives each year than cancer and chronic respiratory disease combined (1). Lifetime risk of CVD approaches 50% for persons age 30 years without known CVD (2). As of 2014, the prevalence of CVD in the US was 36.6%. By 2035, 45.1% of the US population is projected to have some form of CVD (3). Many patients diagnosed with CVD are eligible for participation in cardiac rehabilitation (CR) programs, which include comprehensive, long-term services involving medical evaluation, supervised exercise, cardiac risk factor modification, education, and counseling. Proven benefits include improvement in exercise capacity, risk factors, medication adherence, control of cardiac symptoms, reduction in recurrent myocardial infarction, improved survival after percutaneous coronary intervention and coronary artery bypass surgery, and improved quality of life (4–8). The safety and efficacy of cardiac rehabilitation have been demonstrated in all patients (9). Despite the benefits and safety, CR utilization rates are low. Reasons for non-participation include other comorbidities (arthritis, chronic obstructive pulmonary disease, diabetes with neuropathy) or disability, polypharmacy, frailty, deconditioning, and other challenges that make participation (or the perception of participation) difficult (10–12).

Alternative or adjunctive forms of rehabilitation to reduce CVD burden and improve health should be considered. Singing is a physical activity that involves many components of the vagal nerves (i.e., the pulmonary efferent fibers and afferent fibers of the recurrent laryngeal nerve) manifested as changes in cardiac autonomic regulation (13, 14). It requires both passive and active breathing, muscle coordination and various breathing techniques, with almost no reliance on mobility or skeletal muscle strength. Limited studies have shown potential for singing interventions in patients with chronic obstructive pulmonary disease—primarily by improved lung function (15, 16). Singing also holds promise for improving gait, vocal function and depression in neurological disorders, such as Parkinson's disease (17, 18). Another study examined the impact of singing in cancer patients and caregivers of cancer patients and found preliminary evidence that singing improves mood state and modulates components of the immune system—even after a single choir rehearsal session (19). However, patients with established CVD have not been studied in this context despite promising findings in other chronic disease populations. Because the cardiovascular and respiratory systems are intimately related and work together to deliver oxygen to all cells in the body, singing would be anticipated to have favorable effects on markers of cardiovascular health, and this is what we aim to investigate in this pilot study.

Two excellent biomarkers of cardiovascular health include non-invasive measures of vascular function, such as fingertip peripheral arterial tonometry (PAT), and heart rate variability (HRV). PAT measurements have significant predictive value for future cardiovascular events (20), and microvascular function can be improved by interventions known to reduce cardiovascular risk (21–23). HRV is the variability between R-R intervals in successive heartbeats and is the result of a complex interaction between respiratory activity and autonomic cardiovascular control between the two branches of the autonomic nervous system (sympathetic and parasympathetic) (24). Measurements of HRV have been found to be powerful predictors of cardiac morbidity and mortality (25, 26).

The aim of this pilot study is to investigate the acute effects of singing on biomarkers of cardiovascular health. Our hypothesis is that cardiac patients will have favorable improvements in vascular endothelial function and heart rate variability, after a single session of singing.

## Methods

### Subject recruitment

All subject-related study activities were performed at the Medical College of Wisconsin campus with IRB approval. Adult patients visiting our cardiology clinics were informed of the study and given an informational flyer by their clinic provider. If the patient contacted the study team, a brief overview of the study was provided over the phone and initial eligibility was determined in the form of a short screening questionnaire, which included inclusion and exclusion criteria. Subjects were ≥18 years of age and willing and able to sign informed consent. Exclusion criteria included: permanent pacemaker or implantable cardioverter defibrillator (ICD), history of atrial fibrillation, Parkinson's disease or tremor, amputated upper extremity, presence of upper-arm fistula, fingernail onychomycosis, pregnancy, current tobacco use, current illicit drug use, current excessive alcohol intake (defined as more than 14 drinks/week for women, more than 28 drinks/week for men) unstable coronary heart disease (active symptoms of chest discomfort), supplemental oxygen use, and non-English speaking. If the subject satisfied the above screening criteria, a copy of the IRB consent form was mailed to them, and a single 90-min visit was scheduled during clinic operating hours. Subjects were advised to remain fasting for a minimum of 3 h prior to the study visit.

### General study design

At the study visit, written informed consent was obtained. Subjects were asked to complete a questionnaire including the following information: age, gender, race/ethnicity, history of tobacco use, alcohol use, frequency and intensity of exercise and any physical limitations, past medical and surgical history including hypertension, coronary artery disease (as evidenced by coronary angiography, history of myocardial infarction, percutaneous coronary intervention, or coronary bypass surgery), diabetes mellitus, heart failure, chronic lung disease, high cholesterol, chronic kidney disease, thyroid disease, peripheral arterial disease, and stroke. Current medications were documented. Baseline vital signs (heart rate, blood pressure, pulse oximetry), weight and height were obtained prior to the music intervention.

### Singing protocol

In a seated position in a private exam room, subjects watched and sang along to a 14-min coaching video created by a music professor, which included vocal warm-up exercises followed by the Star-Spangled Banner, repeated at various tempos and pitches. During this video, the professor played the piano and coached the subject through the warm-up and singing. Lyrics were displayed along the bottom of the video.

### Peripheral vascular function assessment using digital peripheral arterial tonometry

Vascular endothelial function was measured by digital pulse arterial tonometry (Endo-PAT 2000, Itamar Medical, Israel). Endothelium-mediated changes in vascular tone after occlusion of the brachial artery are reflecting a downstream hyperemic response, which is a measure for arterial endothelial function (27). Measurements were performed prior to and 1 min following the 14-min singing protocol, according to the manufacturer's instructions (28). Briefly, the subjects were in a supine position for at least 20 min, in a quiet, temperature-controlled room (70–75°F) with a non-condensing humidity. Subjects were asked to remain still and silent during the measurement period with disposable, pneumatic probes on both index fingers. Each recording consisted of 5 min of baseline measurement, 5 min of occlusion measurement, and 5 min post-occlusion measurement (hyperemic period). Occlusion of the brachial artery was performed on the non-dominant upper arm and verified by the absence of a PAT signal from the occluded arm. The occlusion pressure was at least 60 mm Hg above the systolic blood pressure (maximum 200 mm Hg).

PAT measurements were expressed as reactive hyperemia index (RHI) and Framingham reactive hyperemia index (fRHI) (29). The RHI is the PAT signal at the 90–150 s post-deflation interval (30). A normal RHI was defined as >1.67; abnormal RHI was ≤1.67 (31, 32). To provide a better double-sided distribution closer to normal distribution, the log transformation of RHI was calculated (LnRHI). Normal LnRHI was defined as >0.51, abnormal LnRHI was ≤0.51. The fRHI is derived from the PAT signal at the 90- to 120-s post-deflation interval and is inversely related to cardiometabolic risk factors (29). The fRHI interval exhibited the strongest correlation to cardiovascular risk in the Framingham Third Generation Cohort participants using a multivariate risk model (29). Because preceding PAT measurements can affect subsequent measurements, a minimum period of 20 min was required between the pre- and post-singing measurements.

### Heart rate variability assessment

To measure heart rate variability, an appropriately sized and Bluetooth-compatible heart rate sensor strap (Polar, Kempele, Finland) was applied to the subject's bare chest. One-minute-long heart rate and HRV recordings were taken before, during (about 10 min into singing the 14-min video) and (1 min) after completion of singing. The data was transmitted to an iPad using the Elite HRV (Asheville, NC) application and recorded on a study data sheet by research personnel. HRV was reported as the standard deviation of R-R (or NN) intervals (SDNN) and the root mean square of the successive differences (RMSSD), based on beat-to-beat differences in R-R intervals.

### Additional measurements

Perceived exertion with singing was reported by study subjects using the Borg Rating of Perceived Exertion (RPE) scale (33). Subjects were education about the scale prior to the singing intervention. The numerical scale ranges from 6 to 20, where 6 means “no exertion at all” and 20 means “maximal exertion”. The Borg RPE is a well-validated, qualitative scale that is routinely used during cardiac treadmill testing to measure physical activity intensity level and is easy for patients to understand (34). The Borg RPE is the preferred method to assess intensity among those individuals who take medications that affect heart rate or pulse due to the scale's ability to capture exertion from central cardiovascular, respiratory, and nervous system functions (35). At the end of the singing intervention, subjects were asked to rate their perceived exertion level using the Borg RPE.

In addition, following the singing intervention, subjects were asked: On a scale of 1–10, how much did you enjoy singing today (where 1 is “not at all” and 10 is “very much enjoyed”)? Two study staff agreed on a subjective rating of each subject's singing effort using a numerical scale of 1–5, with 1 indicating “very little effort” and 5 as “maximal effort” (see Supplementary Table 1 for more details). The study subjects were blinded to the effort rating.

### Statistical analysis

RHI by digital pulse arterial tonometry was the primary outcome of interest. For a 0.05 range in the RHI with 0.1 standard deviation, the effect size is 0.5. Our a priori power calculation determined that we would need 42 subjects to detect an effect size of 0.5 at α = 0.05 significance level with 90% power. Generalized linear regression models were constructed to determine the effect of covariates (age, gender, history of CAD, prior tobacco use, history of heart failure, hypertension, statin medication use, diabetes, and beta blocker or calcium channel blocker use) on baseline RHI, fRHI, LnRHI, RMSSD, and SDNN.

Paired *t*-tests and repeated measures of variance were used to compare serial measures of vascular function and heart rate variability, respectively. Regression models were also performed to compare pre- and post-outcome measures, while adjusting for baseline values, to determine significant (covariate) predictors of change. In a separate analysis, models were adjusted for baseline RHI as a categorical variable (abnormal RHI ≤ 1.67 or normal RHI > 1.67). Linear mixed models were adjusted for repeated measures. Chi-square and Wilcoxon rank-sum tests were used to compare BORG RPE, self-reported enjoyment, and observed effort on improvement (yes or no) in vascular function and HRV outcome measures. *P*-values < 0.05 were considered significant.

## Results

A total of 379 patients were informed of the study by their cardiologist. Of these, 63 patients had ≥1 exclusion criterion; 119 were not interested in participating (and were not approached by the study team). Of 215 interested in hearing more about the study, the most common reasons for non-participation were inability of study team to get in contact with the patient, travel distance to the hospital, and change of mind after hearing more about the study protocol. Subject characteristics are displayed in Table 1. Sixty subjects were enrolled (mean age 61 ± 13 years, 68% women). Hypertension was the most prevalent cardiovascular disease (CVD) risk factor, found in 60% of subjects, and 55% of subjects were currently on a statin medication. Forty-three percent of subjects had established coronary artery disease (as evidenced by obstructive disease on coronary angiography, history of myocardial infarction, percutaneous coronary intervention, or coronary bypass surgery). Eight subjects did not have one or both measures of RHI secondary to a weak or noisy PAT signal. Two subjects did not have usable HRV data secondary to technical difficulties with equipment.

**Table 1:** Baseline characteristics of participants (*n* = 60).

Age (range)	61 ± 13 (24–90)
Women (%)	41 (68)
Race
% White	46 (77)
% Black	13 (22)
% Asian	1 (2)
BMI (kg/m^2^)	32 ± 8
Prior tobacco use	18 (30)
Known coronary artery disease	26 (43)
History of congestive heart failure	10 (17)
Hypertension	36 (60)
Diabetes mellitus	14 (25)
Current statin use	33 (55)
Prior stroke/TIA	6 (10)
Chronic respiratory disease	17 (28)
Chronic physical or orthopedic limitations	26 (43)
ASCVD risk factors
≤ 2 Risk factors	21 (35)
Known ASCVD or >2 risk factors	39 (65)
Baseline RHI	
Normal > 1.67	34 (57)
Abnormal ≤ 1.67	26 (43)

*Age and BMI shown in mean years ± standard deviation (range). Other values shown as n (%)*.

*RHI, reactive hyperemia index*.

**Figure 1:**
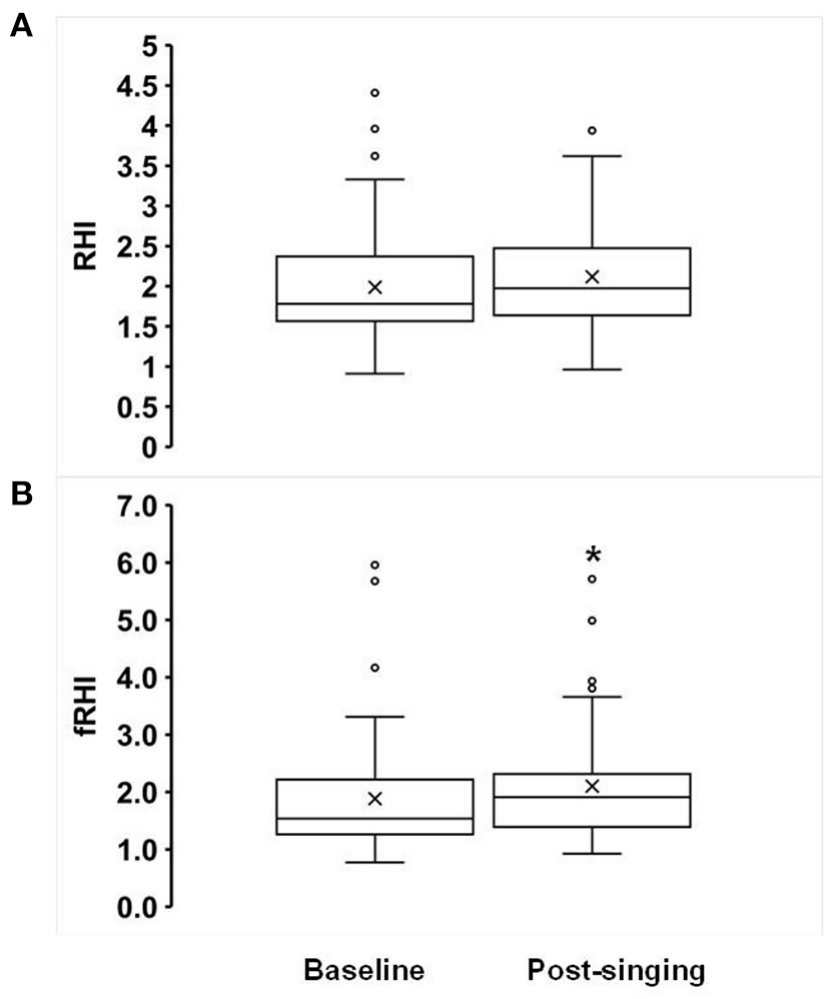
Effects of singing on Reactive Hyperemia Index (RHI, **A**) and Framingham RHI (fRHI, **B**). Box plots showing the following values: the mean (x), median, upper quartile (Q3), lower quartile (Q1), minimum and maximum whiskers as well as outliers. *p < 0.05.

**Figure 2:**
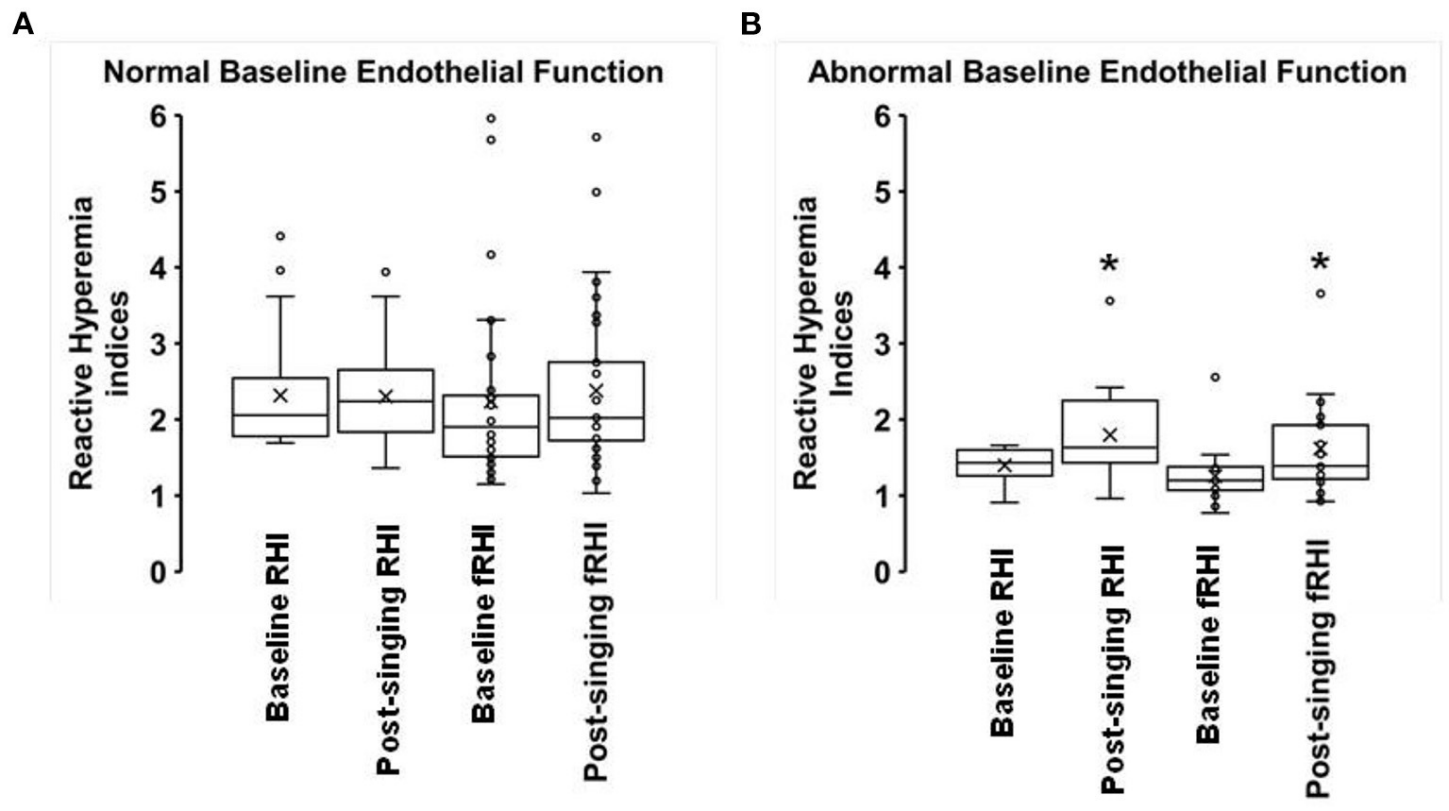
Baseline endothelial function and reactive hyperemia response **(A)** normal baseline, **(B)** *n* = 34; abnormal baseline, *n* = 26. Box plots showing the following values: the mean (x), median, upper quartile (Q3), lower quartile (Q1), minimum and maximum whiskers as well as outliers. *p < 0.05.

Fifty-seven percent of subjects had abnormal baseline endothelial function (defined as an RHI ≤ 1.67). Statin use was associated with lower baseline RHI (β = −0.40 ± 0.2, *p* = 0.041). Statin use and diabetes were associated with lower baseline fRHI (β = −0.62 ± 0.3, *p* = 0.015 and −0.63 ± 0.3, *p* = 0.006, respectively). Results are displayed in Table 2. Although there was a trend toward improvement in the RHI after singing, this was not statistically significant (1.99 ± 0.10 to 2.12 ± 0.09, *p* = 0.17, Figure 1A). There was a significant increase in the fRHI after singing (1.88 ± 0.14 to 2.10 ± 0.14, *p* = 0.023, Figure 1B). Subjects with abnormal baseline endothelial function demonstrated significant improvement in the RHI after singing (1.40 ± 0.05 to 1.80 ± 0.13, *p* = 0.01, Figure 2B) compared to subjects with normal baseline RHI (2.32 ± 0.12 to 2.30 ± 0.11, *p* = 0.82, Figure 2A), with a similar result in the fRHI. Similarly, subjects at higher ASCVD risk, defined as established ASCVD or >2 risk factors, demonstrated a significant improvement in fRHI (1.82 ± 0.14 to 2.08 ± 0.13, *p* = 0.015) compared to subjects at lower ASCVD risk (2.35 ± 0.73 to 2.28 ± 0.70, *p* = 0.78). When stratified by gender, women (68% of cohort) had a statistically significant improvement in fRHI (1.80 ± 0.9 to 2.12 ± 0.9, *p* = 0.007) with singing when compared to males (2.04 ± 1.3 to 2.07 ± 1.2, *p* = 0.83). However, after adjustment for baseline fRHI, gender was no longer significant. The presence of abnormal RHI ≤ 1.67 at baseline consistently predicted improvement with singing after adjustment for pre-specified covariates (age, gender, history of CAD, hypertension, diabetes, history of heart failure, statin use; β = 0.38 ± 0.18 to 0.43 ± 0.18, *p* < 0.05).

**Table 2:** Results (*n* = 60).

	**Baseline**	**Singing**	**Post-singing**
**Vascular function**			
RHI	1.99 ± 0.10	-	2.12 ± 0.09
fRHI	1.88 ± 0.14	-	*2.10 ± 0.14
LnRHI	0.63 ± 0.05	-	0.70 ± 0.04
**HRV**			
RMSSD	42.0 ± 4.9	*32.6 ± 4.0	40.4 ± 4.9
SDNN	54.0 ± 4.8	*33.5 ± 2.8	*42.9 ± 4.0
Average HR (bpm)	68.9 ± 1.8	70.3 ± 1.9	*66.8 ± 1.8
**Blood pressure (mmHg)**			
MAP	91 ± 1.3	*96 ± 2.2	*93 ± 1.3
SBP	128 ± 1.9	*136 ± 2.0	*131 ± 2.1
DBP	73 ± 1.3	*78 ± 1.5	74 ± 1.3
Oxygen saturation (%)	96.3 ± 0.27	*97.5 ± 0.22	96.0 ± 0.77
Enjoyment level	-	-	7.4 (1–10)
Observed effort	-	-	3.7 (1–5)
BORG RPE score	-	-	8.9 (6–14)

^*^p < 0.05 when compared to baseline.

*Values represent means ± standard error or (range)*.

*RHI, reactive hyperemia index; fRHI, Framingham reactive hyperemia index; LnRHI, log-transformed reactive hyperemia index; HRV, heart rate variability; RMSSD, root mean square of the successive differences; SDNN, standard deviation of R-R (or NN) intervals; HR, heart rate; MAP, mean arterial blood pressure; SBP, systolic blood pressure; DBP, diastolic blood pressure; RPE, rating of perceived exertion*.

**Figure 3:**
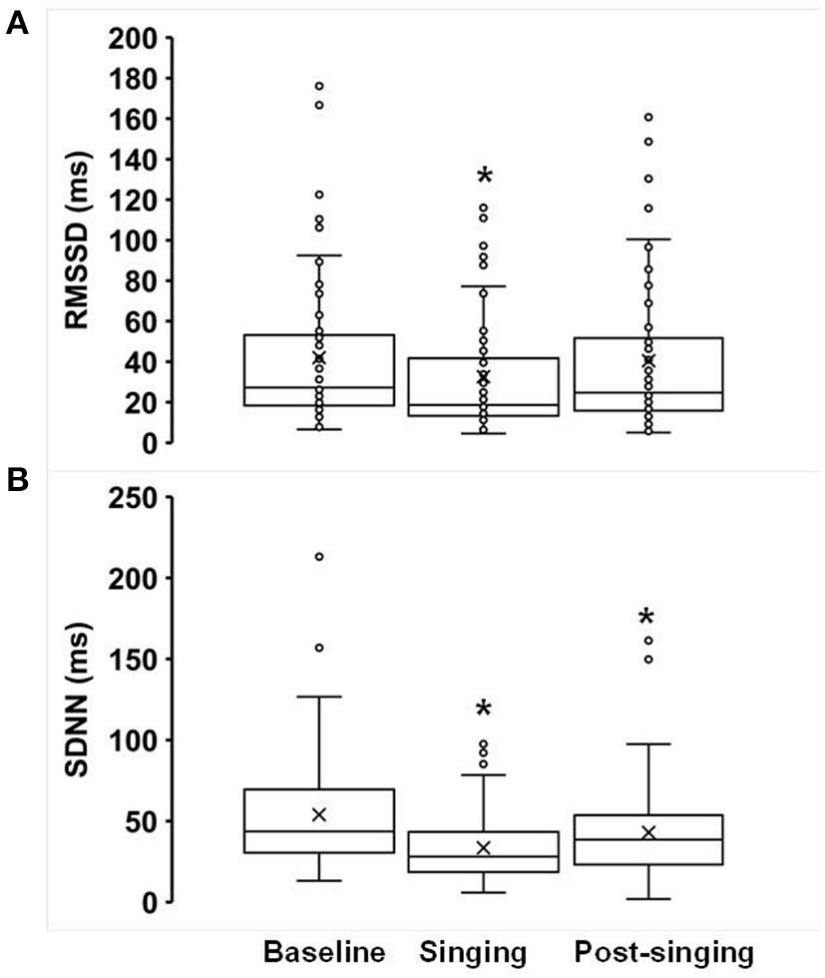
Effects of singing on heart rate variability **(A)** RMSSD and **(B)** SDNN. Box plots showing the following values: the mean (x), median, upper quartile (Q3), lower quartile (Q1), minimum and maximum whiskers as well as outliers. *p < 0.05.

There was a significant decrease in both time-domain measures of HRV during singing: RMSSD decreased from 42 ± 4.9 ms to 33 ± 4.0 ms (*p* = 0.004, Figure 3A), and SDNN decreased from 54 ± 4.8 ms to 34 ± 2.8 ms (*p* < 0.001, Figure 3B). In addition, there was a reduction in SDNN post-singing (43 ± 4.0) compared to baseline (*p* = 0.014, Figure 3B). The mean heart rate (averaged over a 1-min recording) decreased pre- to post-singing (69 ± 2 to 67 ± 2 beats per min, *p* = 0.008, Supplementary Figure 1). The mean heart rate during singing (70 ± 2 bpm) was not different from baseline (*p* = 0.16). The oxygen saturation, as measured by pulse oximetry, increased while singing compared to baseline (97.5 ± 0.2% from 96.3 ± 0.3%, *p* < 0.0001). Mean arterial blood pressure (MAP, mm Hg) increased during (95.8 ± 2.2) and post-singing (93.3 ± 1.3) when compared to baseline [(91.5 ± 1.3), all *p* ≤ 0.02].

**Figure 4:**
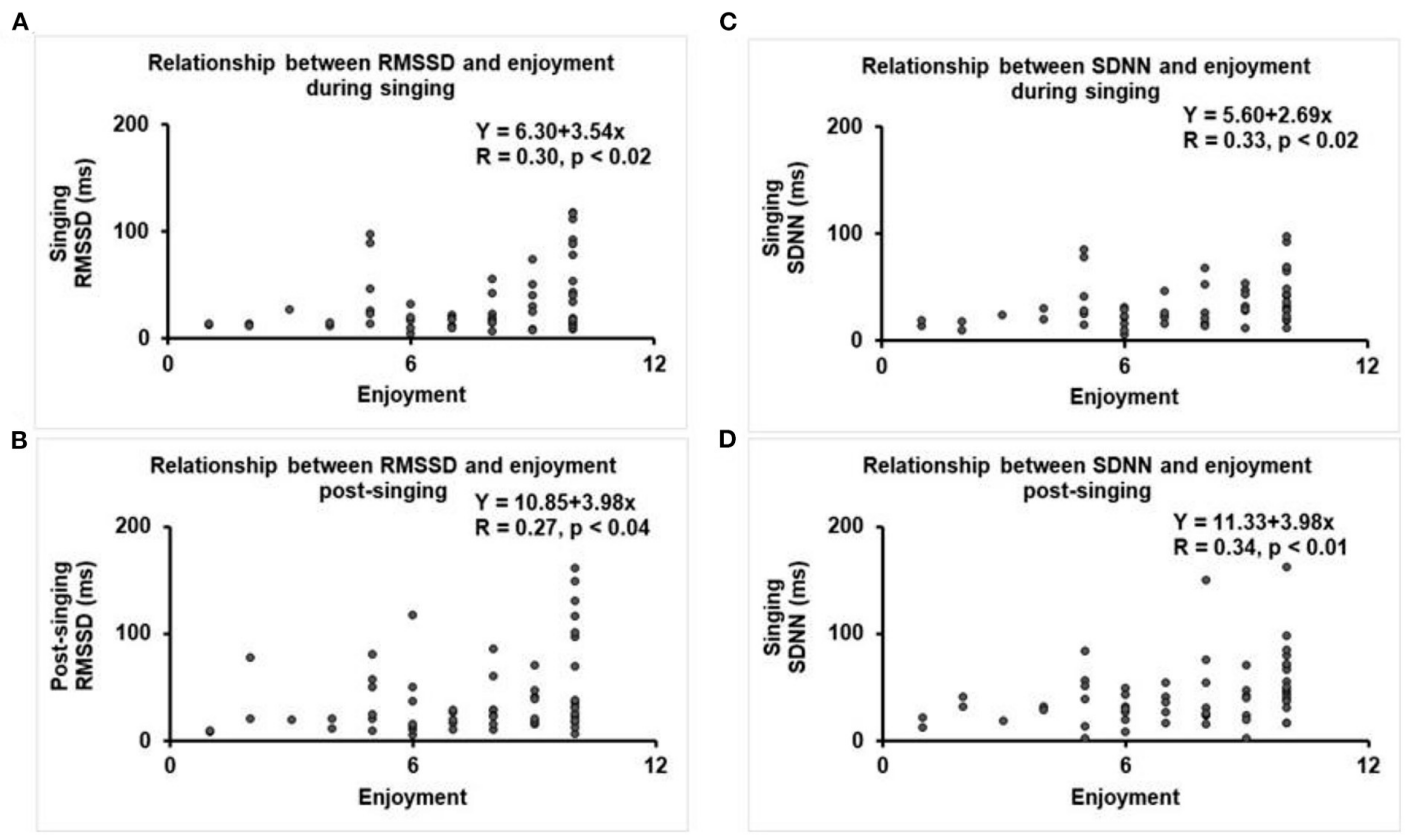
Regression plots between HRV and level of enjoyment (scale 1–10). **(A)** RMSSD during singing, **(B)** SDNN during singing, **(C)** RMSSD after singing, and **(D)** SDNN after singing.

Regression analyses were performed with HRV as the dependent variable and level of enjoyment (scale 1–10) as the independent variable. There were low-moderate, but statistically significant, positive correlations between both RMSSD and SDNN and enjoyment level during and after the singing intervention (Figure 4). There was no correlation between achieved BORG RPE score, level of enjoyment, or observed singing effort with the reactive hyperemic response (data not shown). In regression models (data not shown), none of the covariates (age, gender, history of CAD, hypertension, statin medication use, diabetes, and beta blocker use) were predictors of change in HRV or vascular function (RHI, LnRHI, fRHI), when adjusted for baseline values.

## Discussion

To our knowledge, this is the first study to demonstrate acute improvement in peripheral endothelial function after a single, short period of singing. Subjects with abnormal baseline endothelial function and/or at highest ASCVD risk demonstrated the most significant improvement in endothelial function with singing when compared to subjects with normal baseline endothelial function and low ASCVD risk. As such, singing as a non-pharmacologic therapeutic may be most beneficial to patients with established atherosclerotic vascular disease.

A small number of prior studies have examined the impact of *listening* to music on markers of vascular health. Ripley et al. (36) randomized 70 subjects undergoing cardiac catherization for suspected coronary artery disease to music listening (slow, relaxing contemporary music) or no music. Vascular endothelial function, as measured by PAT, was performed before and after catheterization. Listening to music did not elicit a vasodilator response, lower blood pressure, or heart rate, and did not relieve anxiety or stress. Patient musical preferences were not considered, and effects of moderate sedation and/or physical discomfort, may have contributed to the null findings. In a separate study, 9 of 10 volunteers experienced increased brachial artery flow-mediated dilation (FMD) (2.7% absolute increase; *p* < 0.001) after listening to 30 min of joyful music (self-selected by the subject). The mean FMD after listening to joyful music was significantly larger than the FMD response to anxiety-provoking music (0.6% absolute decrease; *p* = 0.005). However, this was a very small study consisting of young, healthy volunteers (37). Our study is larger with older subjects, many with established cardiovascular disease, and examines the impact of active singing rather than passive listening, on vascular health. In our study, active singing did not significantly improve endothelial function in healthy subjects with normal baseline endothelial function. These data suggest that subjects with healthier endothelium at baseline may need a larger stimulus to affect change or cannot augment their vascular endothelial function further. Vascular function remains an important biomarker to include in future studies since a meta-analysis concluded an 8–13% lower risk of cardiovascular events per 1%-point increase in brachial artery FMD (38).

Our study included 68% women. When stratified by gender, women had a statistically significant improvement in the fRHI (*n* = 41) compared to men (*n* = 19). Women had a lower mean baseline fRHI (1.80 ± 0.9 vs. 2.04 ± 1.3 for men, *p* = NS), and the gender difference was no longer significant after adjustment for baseline fRHI. Our pilot study showed significantly more interest in (singing) participation by women. This is noteworthy since gender disparities in cardiac rehabilitation referral and adherence favor men even though women completing cardiac rehabilitation may experience greater reductions in mortality (39, 40). Singing could serve as a more appealing therapy incorporated into traditional cardiac rehabilitation for older women (41). Moreover, whether singing as a component of cardiac rehabilitation could reduce barriers to referral and adherence to cardiac rehabilitation participation in women would be important to study in future trials.

Another means by which music may benefit the cardiovascular systems is by way of cardiac autonomic regulation. HRV reflects an interplay between the sympathetic and parasympathetic branches of the autonomic nervous system. Overall, systematic reviews demonstrate a positive impact of music listening on HRV, suggesting enhanced parasympathetic activity (42, 43). Far fewer studies have examined singing as a musical intervention. Singing and instrument playing, which are more active interventions, probably have different effects on cardiovascular physiology, as supported by the present study.

We examined measures of HRV prior to, during and after singing. The pattern of HRV changes observed were similar to those seen with exercise activity whereby exercise elicits a reduction in HRV when expressed in the time domain (SDNN and RMSSD), followed by a recovery toward baseline HRV post-exercise. The reduction in HRV during exercise is thought to be primarily mediated by reduced cardiac parasympathetic neural activity (cPNA), i.e., “parasympathetic withdrawal”. The relative autonomic balance shifts from predominantly “parasympathetic control” at rest and with low exercise intensities to mainly “sympathetic control” at higher intensities. Loading of cardiopulmonary baroreceptors (due to increase in venous return from muscle pump action), muscle mechanoreceptors and systemic sympatho-adrenal activation are thought to also have roles in HRV changes associated with exercise (44). However, the physical act of singing has little to no reliance on any skeletal muscle activity. With singing, pulmonary receptors, lung mechanical effects and the adrenal response may be the drivers of “parasympathetic withdrawal”. For example, respiratory sinus arrhythmia (RSA) occurs when a person's heart rate relates to their breathing cycle. Typically, the heart rate increases with inspiration and decreases during expiration (increasing overall HRV). Prior studies have shown that efficiency of pulmonary gas exchange is improved by RSA. Evidence is accumulating of a possible dissociation between RSA and vagal control of the heart rate, suggesting differential controls between respiratory modulation of cardiac vagal outflow and cardiac vagal tone (45). Evaluating the adrenal response to singing, perhaps with salivary cortisol levels, may help to elucidate contributions to “parasympathetic withdrawal” related to adrenal activation (or deactivation), and this should be considered in future studies. Additionally, examining HRV in both the time and frequency domains can provide additional insight to the relative contributions of both the sympathetic and parasympathetic nervous systems.

We did see a modest, positive correlation between self-reported level of enjoyment with the singing intervention and HRV response. While this doesn't capture the subjects' musical preferences, it may serve as a rough surrogate for individual emotional valence (pleasure or displeasure). A narrative review of more than 1,300 subjects with 29 independent studies showed that the majority of studies did not consider music selection (43). Published studies to-date suffer from poor control of individual music preferences. Individual responses to music can be influenced by personal preferences, familiarity with music, environment, prior music experience, and other health factors (42). Furthermore, the construct of singing is complex. Singing happens in social contexts which complicates an objective assessment of the effects of singing itself. Other factors, including vocal contagion, social cohesion, alterations in breathing, other physiological changes, and song structure further add to this complexity (14, 46).

The limitations of this pilot study include the non-randomized, unblinded trial design, lack of a control arm, lack of consideration to personal music preferences with the intervention, and HRV measurements at only 1-min increments in the time domain. Some studies recommend HRV recordings longer than 2 min for improved accuracy (47). The recommended 3-h fast may be insufficient to exclude post-prandial vascular function measurements. Fortunately, almost all the study subjects had an overnight fast (at least 8 h) since the study visit was scheduled in advance and performed in the morning. Offsetting these weaknesses are strengths of this study including the active singing (rather than passive listening) music intervention, the high percentage of women enrolled, and a study population with known cardiovascular disease or risk factors.

## Conclusions

A short duration of singing improves peripheral vascular function acutely in older adults, particularly those with established ASCVD or at high ASCVD risk. The HRV, blood pressure, and oxygen saturation responses to acute singing mirror the effects of light-intensity exercise. While the acute vascular response to a single bout of singing may be predictive of the vascular adaptation to longer and more sustained singing interventions, this remains to be confirmed in large, randomized trials. The promising effects of music interventions on cardiovascular physiology, coupled with the low cost and safety, warrant further exploration in subjects with cardiovascular disease.

## Data availability statement

The raw data supporting the conclusions of this article will be made available by the authors, without undue reservation.

## Ethics statement

The studies involving human participants were reviewed and approved by Medical College of Wisconsin. The patients/participants provided their written informed consent to participate in this study. Written informed consent was not obtained from the minor(s)' legal guardian/next of kin for the publication of any potentially identifiable images or data included in this article.

## Author contributions

JK and LT conceptualized the study design, obtained pilot funding, and executed the study. TR created the music video used for the singing intervention and was also involved in the study's conception. AV, MF, and EO performed data analyses and constructed graphs, plots, tables, and under the supervision of JK. JK and KS wrote the manuscript. MW provided vascular expertise and contributed to major manuscript revisions. All authors have reviewed and read and agreed to the published version of the manuscript.

## Funding

This pilot research project was funded by the Medical College of Wisconsin Research Affairs Committee. This publication was supported in part by the National Center for Complementary & Integrative Health of the National Institutes of Health under Award Number R33AT010680.

## Conflict of interest

The authors declare that the research was conducted in the absence of any commercial or financial relationships that could be construed as a potential conflict of interest.

## Publisher's note

All claims expressed in this article are solely those of the authors and do not necessarily represent those of their affiliated organizations, or those of the publisher, the editors and the reviewers. Any product that may be evaluated in this article, or claim that may be made by its manufacturer, is not guaranteed or endorsed by the publisher.

## Supplementary material

Supplementary Figure 1 Heart rate patterns before, during, and after singing **(A)** minimum heart rate, **(B)** average heart rate, **(C)** maximum heart rate. Box plots showing the following values: the mean (x), median, upper quartile (Q3), lower quartile (Q1), minimum and maximum whiskers as well as outliers. **p* < 0.05.

Supplementary Table 1 Observed singing effort rating.
