# Supplementary material for: Acute effects of singing on cardiovascular biomarkers
Source: Front Cardiovasc Med. 2022 Jul 18;9:869104. doi: 10.3389/fcvm.2022.869104 (PMC9339901; doi:10.3389/fcvm.2022.869104)

10.3389/fcvm.2022.869104

Supplementary Table 1. Observed singing effort rating

Numerical Rating	Effort	Examples
1	Very little	Talking and/or not following lyrics
2	Light	Singing some lyrics and/or singing with a quiet voice
3	Some	Singing and hitting most of the lyrics with singing or humming
4	Moderate	Singing lyrics throughout entire video
5	Maximal	Singing loudly, very focused, and following all lyrics well

10.3389/fcvm.2022.869104

Supplementary Figure 1. Heart rate patterns before, during, and after singing

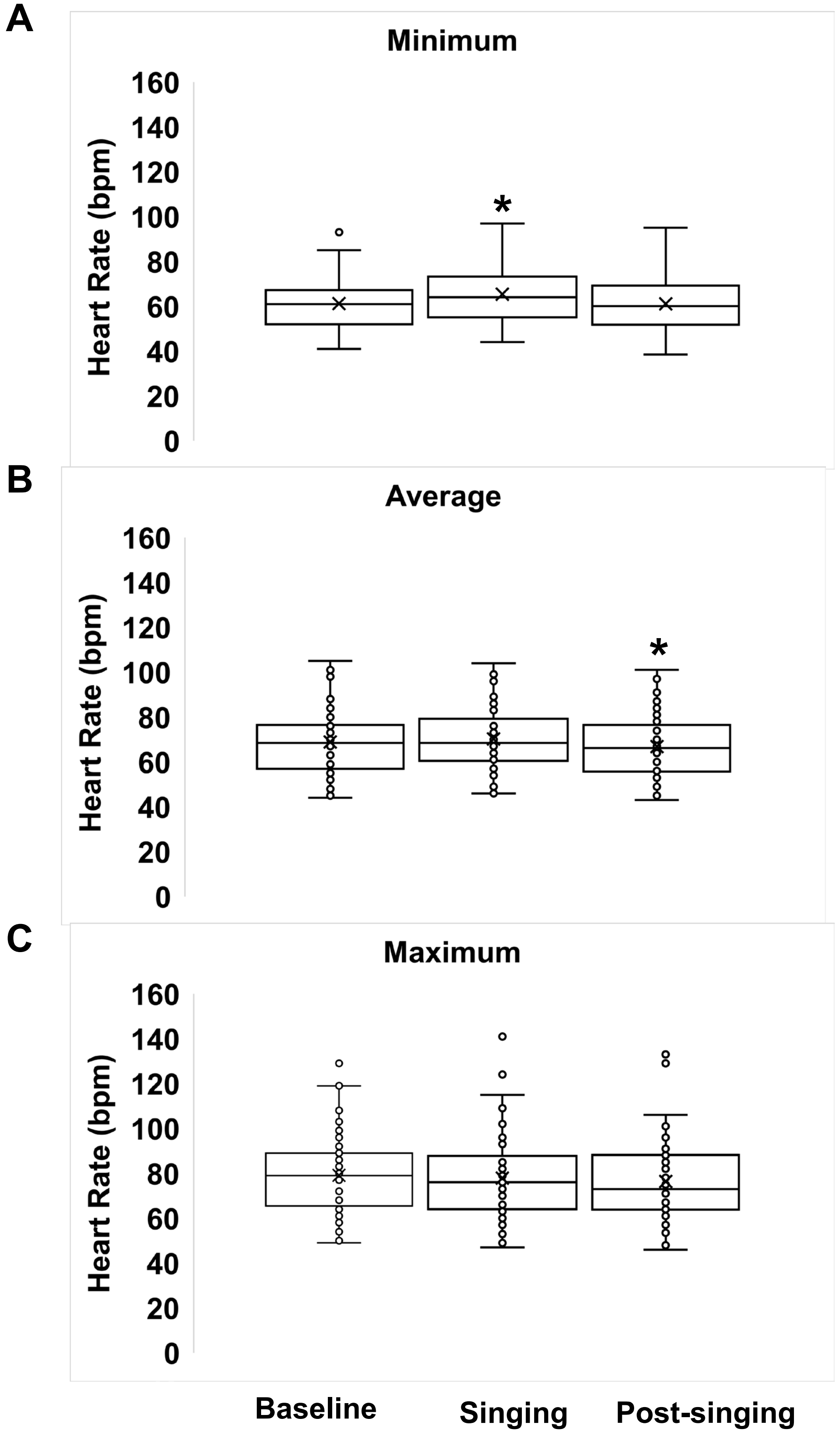

Supplement: Supplementary Figure 1 — Heart rate patterns before, during, and after singing (A) minimum heart rate, (B) average heart rate, (C) maximum heart rate. Box plots showing the following values: the mean (x), median, upper quartile (Q3), lower quartile (Q1), minimum and maximum whiskers as well as outliers. *p < 0.05. [file Data_Sheet_1.pdf]
